# Supplementary material for: A UMLS-based spell checker for natural language processing in vaccine safety
Source: BMC Med Inform Decis Mak. 2007 Feb 12;7:3. doi: 10.1186/1472-6947-7-3 (PMC1805499; doi:10.1186/1472-6947-7-3)
Supplement: Additional file 13 — RAP application directory. Sets up directory for RAP (RDF application for PHP) source code [file 1472-6947-7-3-S13.gz › rap/doc/phpdoc/resModel/ResSeq.html]

xml version="1.0" encoding="iso-8859-1"?


Docs For Class ResSeq


## Class ResSeq

Description

Description |
Methods (details)

This interface defines methods for accessing RDF Sequence resources.

These methods operate on the RDF statements contained in a model.

History:

- access: public
- author: Daniel Westphal <at d-westphal dot de mail at d-westphal dot de>
- version: V0.9.1

Located in /resModel/ResSeq.php (line 19)

```
Object
   |
   --Node
      |
      --Resource
         |
         --ResResource
            |
            --ResContainer
               |
               --ResSeq
```

Method Summary

Description |
Methods (details)

ResSeq
ResSeq
([string $uri = null])

boolean
addAtIndex
(integer $index, mixed $object, object ResResource/ResLiteral $resResource)

object ResResource/ResLiteral
getMember
(integer $index)

integer
indexOf
(object ResResource/ResLiteral $object)

void
removeAtIndex
(integer $index)

boolean
set
(integer $index, mixed $object)

Methods

Description |
Methods (details)

Constructor ResSeq (line 29)

Constructor You can supply a URI

- access: public

ResSeq

ResSeq
([string $uri = null])

- string
  $uri

addAtIndex (line 45)

Insert a new member into the sequence at the specified position.

The existing member at that position, and all others with higher indexes, have their index increased by one.

- access: public

boolean

addAtIndex
(integer $index, mixed $object, object ResResource/ResLiteral $resResource)

- integer
  $index
- object ResResource/ResLiteral
  $resResource

getMember (line 75)

Get the member at a given index

- access: public

object ResResource/ResLiteral

getMember
(integer $index)

- integer
  $index

indexOf (line 98)

Return the index of a given member of the sequence.

If the same value appears more than once in the sequence, it is undefined which of the indexes will be returned. If the member is not found in this sequence, a value of 0 is returned.

- access: public

integer

indexOf
(object ResResource/ResLiteral $object)

- object ResResource/ResLiteral
  $object

removeAtIndex (line 122)

Remove the member at the specified index.

All other members with a higher index will have their index reduced by one.

- access: public

void

removeAtIndex
(integer $index)

- integer
  $index

set (line 146)

Set the value at a given index in the sequence.

If the index is not in the range of the sequence, false is returned

- access: public

boolean

set
(integer $index, mixed $object)

- integer
  $index

#### Inherited Methods


Inherited From ResContainer

> ResContainer::ResContainer()  
> ResContainer::add()  
> ResContainer::contains()  
> ResContainer::getMembers()  
> ResContainer::isAlt()  
> ResContainer::isBag()  
> ResContainer::isSeq()  
> ResContainer::remove()  
> ResContainer::size()

Inherited From ResResource

> ResResource::ResResource()  
> ResResource::addProperty()  
> ResResource::equals()  
> ResResource::getAssociatedModel()  
> ResResource::getIsAnon()  
> ResResource::getProperty()  
> ResResource::hasProperty()  
> ResResource::listProperties()  
> ResResource::removeAll()  
> ResResource::removeProperties()  
> ResResource::setAssociatedModel()  
> ResResource::setIsAnon()  
> ResResource::setURI()

Inherited From Resource

> Resource::Resource()  
> Resource::equals()  
> Resource::getLabel()  
> Resource::getLocalName()  
> Resource::getNamespace()  
> Resource::getURI()  
> Resource::toString()

Inherited From Object

> Object::toString()

Documentation generated on Thu, 7 Jul 2005 13:43:52 +0200 by phpDocumentor 1.3.0RC3
